# Supplementary material for: Intracellular carbon storage by microorganisms is an overlooked pathway of biomass growth
Source: Nat Commun. 2023 Apr 19;14:2240. doi: 10.1038/s41467-023-37713-4 (PMC10115882; doi:10.1038/s41467-023-37713-4)
Supplement: Supplementary file 1 — Supplmentary information [file 41467_2023_37713_MOESM1_ESM.pdf]

Supplementary materials for:

Intracellular carbon storage by microorganisms is an overlooked pathway of biomass growth

Kyle Mason-Jones*[1,2], Andreas Breidenbach[2,3], Jens Dyckmans[4], Callum C. Banfield[2,3], Michaela A. Dippold§[2,3]

* k.masonjones@nioo.knaw.nl

§ michaela.dippold@uni-tuebingen.de

1 Department of Terrestrial Ecology, Netherlands Institute of Ecology (NIOO-KNAW), 6708 PB Wageningen, the Netherlands

2 Biogeochemistry of Agroecosystems, Department of Crop Sciences, Georg-August University of Göttingen, 37075 Göttingen, Germany

3 Geo-Biosphere Interactions, Department of Geosciences, University of Tübingen, 72076 Tübingen, Germany.

4 Centre for Stable Isotope Research and Analysis, Georg-August University of Göttingen, 37077 Göttingen, Germany.

Supplementary A: Supplementary figures

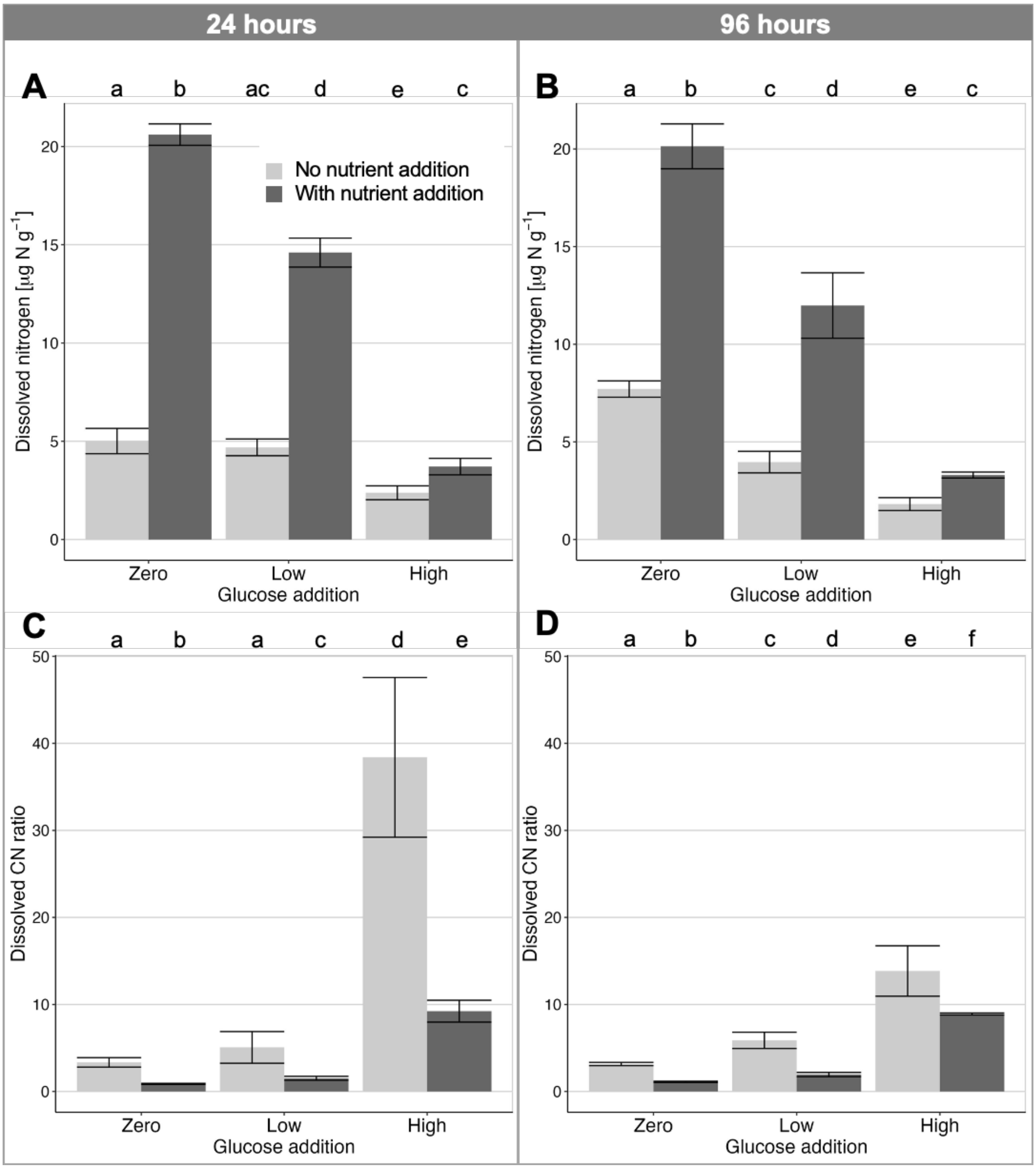

Fig. S1 Dissolve nitrogen and C:N ratio. Dissolved nitrogen after 24 h (**A**) and 96 h (**B**) following addition of glucose at 0, 90 and 400 µg C g^{-1} soil (Zero, Low, High) with or without mineral nutrient supply (N, P, K, S), and the corresponding dissolved C:N ratio (dissolved organic C to total dissolved N) at the corresponding timepoints (**C** and **D**). Error bars show mean±standard deviation, with n = 4 independent soil microcosms. Different letters above the plots indicate significant differences with $p < 0.05$ (2-sided Tukey HSD test on log-transformed values, which adjusts for multiple comparisons).

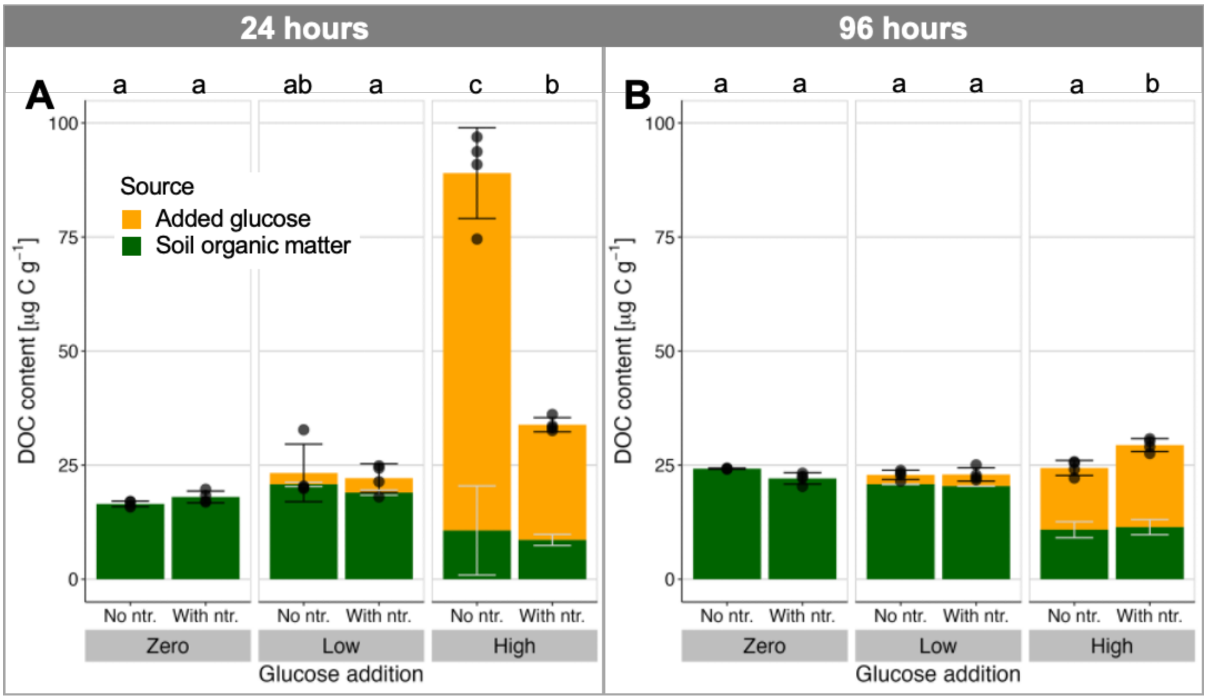

Fig. S2 Dissolved organic carbon. Organic carbon extractable into 0.5 M K_2SO_4 after 24 h (**A**) and 96 h (**B**) following addition of glucose at 0, 90 and 400 µg C g^{-1} soil (Zero, Low, High) with or without mineral nutrient supply (N, P, K, S). Contrasting colours reflect the source of the extractable biomass as determined by isotopic composition, with light gray error bars showing mean±standard deviation of the relative composition. Black error bars show mean±standard deviation of the total. Different letters above the plots indicate significant differences in total DOC with $p < 0.05$ (2-sided Tukey HSD test on log-transformed values, which adjusts for multiple comparisons), with n = 4 independent soil microcosms.

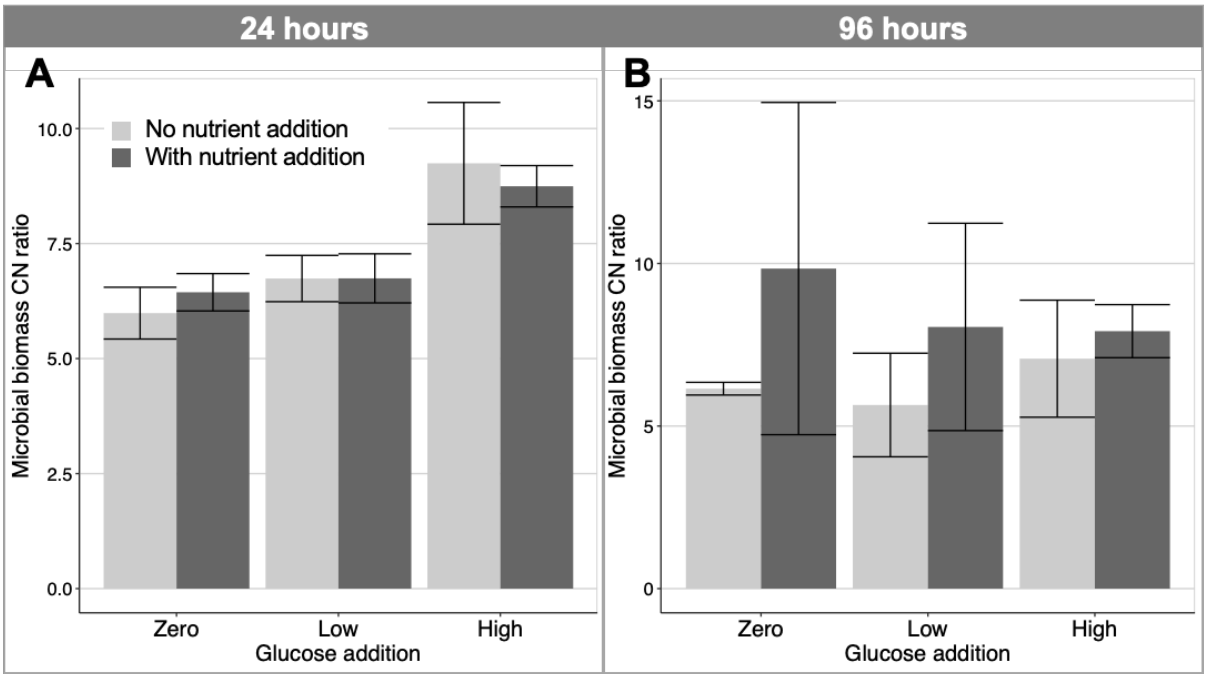

Fig. S3 C:N ratio of extractable microbial biomass. (**A**) 24 h and (**B**) 96 h after glucose and/or nutrient addition to soil microcosms. Error bars show mean±standard deviation, with n = 4 independent soil microcosms, except for one treatment n = 3 (zero glucose, no nutrients at 96 h).

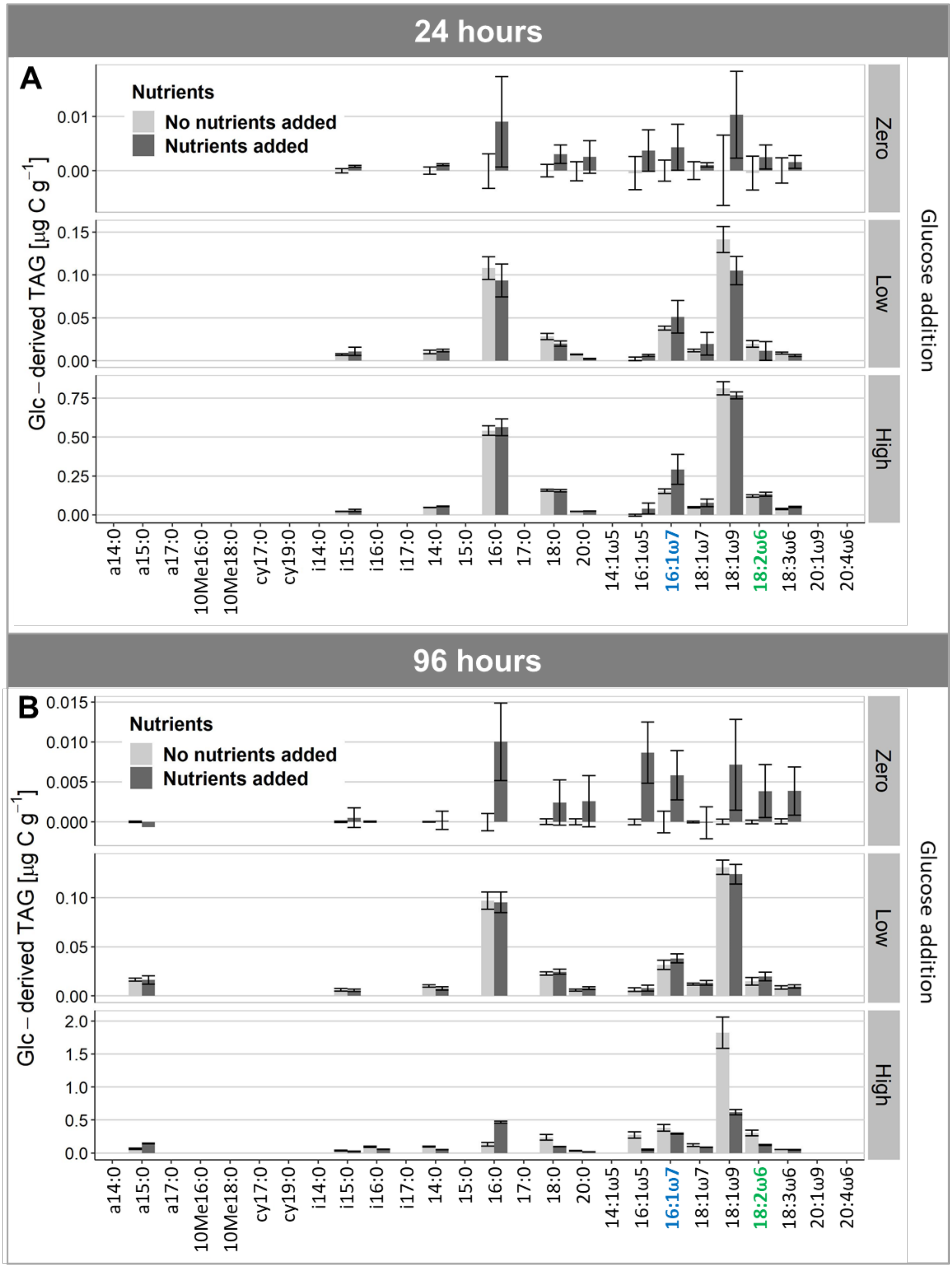

Fig. S4 Fatty acid profile of glucose-derived TAGs. (**A**) 24 h and (**B**) 96 h following addition of glucose at 0, 90 and 400 µg C g^{-1} soil (Zero, Low, High) with or without mineral nutrient supply (N, P, K, S). The diagnostic bacterial biomarker 16:1ω6 (highlighted blue on the horizontal axis) and fungal biomarker 18:2ω6 (highlighted in green) showed substantial incorporation of glucose-derived C. Error bars show mean±standard deviation, with n = 4 independent soil microcosms. Note that the vertical axis scale varies between glucose treatments.

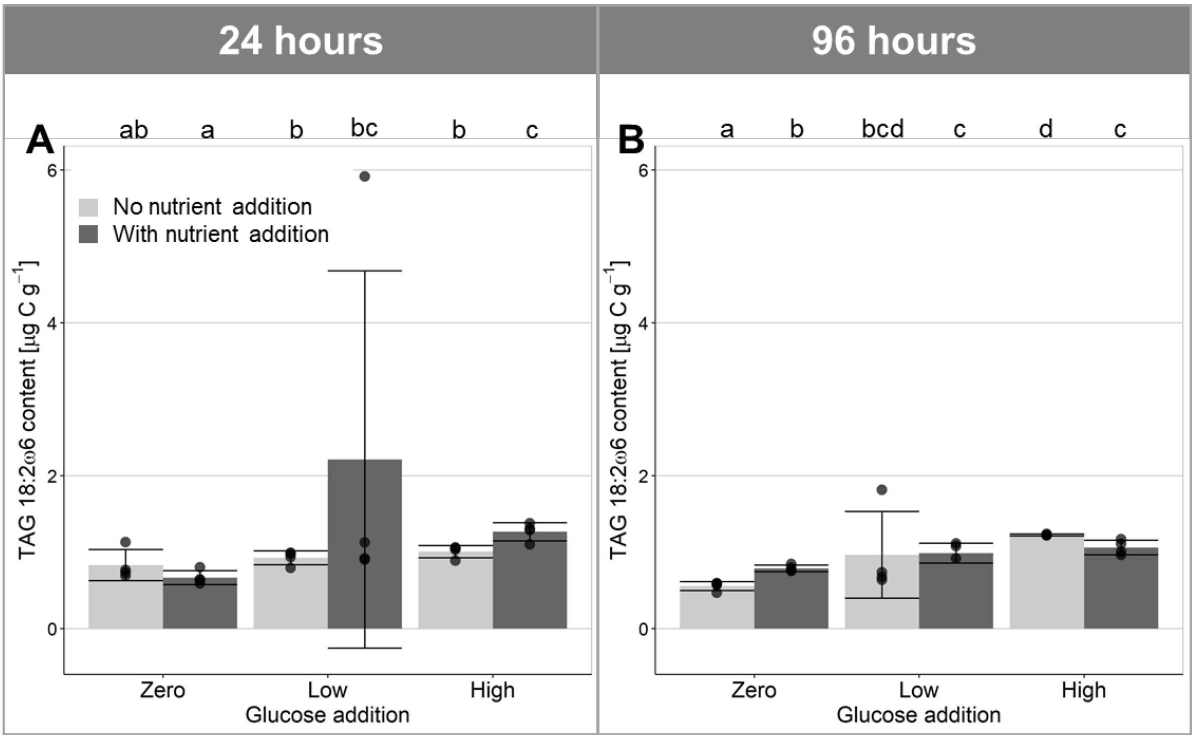

Fig. S5 Total soil content of fungal biomarker TAG 18:2ω6. (**A**) 24 h and (**B**) 96 h following addition of glucose at 0, 90 and 400 µg C g^{-1} soil (Zero, Low, High) with or without mineral nutrient supply (N, P, K and S). Error bars show mean±standard deviation, with n = 4 independent soil microcosms. Lowercase letters above the plots show post-hoc differences with $p<0.05$ (2-sided pairwise comparison of medians with Benjamini-Hochberg adjustment for multiple comparisons).

Supplementary B: Two-pool isotope mixing model

The fraction of O atoms in DNA originating from the added $H_2{}^{18}O$ was calculated using a standard two-pool isotope mixing model[1], which is derived from mass-balance principles:

$$O_{add} = \frac{(atom\%_{tot} - atom\%_{nat})}{(atom\%_{add} - atom\%_{nat})}$$

where

O_{add} is the fraction of O in DNA that originated from the added (^{18}O-labelled) water

$atom\%_{tot}$ is the isotopic abundance of ^{18}O in the total extracted DNA (in atom percent)

$atom\%_{add}$ is the ^{18}O isotopic abundance of soil water during the incubation (in atom percent)

$atom\%_{nat}$ is the ^{18}O isotopic abundance of DNA in the absence of ^{18}O-labelled water (i.e. the natural ^{18}O abundance in DNA from the experimental soil prior to labelling)

Reference:

1. Mason-Jones, K., Schmücker, N. & Kuzyakov, Y. Contrasting effects of organic and mineral nitrogen challenge the N-Mining Hypothesis for soil organic matter priming. *Soil Biol. Biochem.* **124**, 38–46 (2018).
