# Supplementary material for: Intracellular carbon storage by microorganisms is an overlooked pathway of biomass growth
Source: Nat Commun. 2023 Apr 19;14:2240. doi: 10.1038/s41467-023-37713-4 (PMC10115882; doi:10.1038/s41467-023-37713-4)
Supplement: Supplementary file 3 — Reporting Summary [file 41467_2023_37713_MOESM3_ESM.pdf]

nature portfolio

Corresponding author(s): Kyle Mason-Jones

Last updated by author(s): Jun 9, 2022

Reporting Summary

Nature Portfolio wishes to improve the reproducibility of the work that we publish. This form provides structure for consistency and transparency in reporting. For further information on Nature Portfolio policies, see our Editorial Policies and the Editorial Policy Checklist.

Statistics

For all statistical analyses, confirm that the following items are present in the figure legend, table legend, main text, or Methods section.

n/a	Confirmed	
☐	☒	The exact sample size (*n*) for each experimental group/condition, given as a discrete number and unit of measurement
☐	☒	A statement on whether measurements were taken from distinct samples or whether the same sample was measured repeatedly
☐	☒	The statistical test(s) used AND whether they are one- or two-sided<br>*Only common tests should be described solely by name; describe more complex techniques in the Methods section.*
☒	☐	A description of all covariates tested
☐	☒	A description of any assumptions or corrections, such as tests of normality and adjustment for multiple comparisons
☐	☒	A full description of the statistical parameters including central tendency (e.g. means) or other basic estimates (e.g. regression coefficient) AND variation (e.g. standard deviation) or associated estimates of uncertainty (e.g. confidence intervals)
☐	☒	For null hypothesis testing, the test statistic (e.g. *F*, *t*, *r*) with confidence intervals, effect sizes, degrees of freedom and *P* value noted<br>*Give P values as exact values whenever suitable.*
☒	☐	For Bayesian analysis, information on the choice of priors and Markov chain Monte Carlo settings
☒	☐	For hierarchical and complex designs, identification of the appropriate level for tests and full reporting of outcomes
☐	☒	Estimates of effect sizes (e.g. Cohen's *d*, Pearson's *r*), indicating how they were calculated

Our web collection on statistics for biologists contains articles on many of the points above.

Software and code

Policy information about availability of computer code

Data collection	Agilent MassHunter GC/MS Acquisition B.07.04.2260, Thermo Fisher Scientific Isodat 2.0
Data analysis	R version 4.1.1. R Core Team (2021). R: A language and environment for statistical computing. R Foundation for Statistical Computing, Vienna, Austria. URL https://www.R-project.org/.<br><br>R Studio for macOS (Version 1.4.1717)

For manuscripts utilizing custom algorithms or software that are central to the research but not yet described in published literature, software must be made available to editors and reviewers. We strongly encourage code deposition in a community repository (e.g. GitHub). See the Nature Portfolio guidelines for submitting code & software for further information.

Data

Policy information about availability of data

All manuscripts must include a data availability statement. This statement should provide the following information, where applicable:

- Accession codes, unique identifiers, or web links for publicly available datasets
- A description of any restrictions on data availability
- For clinical datasets or third party data, please ensure that the statement adheres to our policy

Data generated during this study, alongside supporting metadata and R scripts for calculations and graphics, will be uploaded onto the Zenodo repository under DOI 10.5281/zenodo.6386047 at the time of manuscript acceptance, with fully open access under a Creative Commons Attribution 4.0 International licence.

Field-specific reporting

Please select the one below that is the best fit for your research. If you are not sure, read the appropriate sections before making your selection.

☒ Life sciences ☐ Behavioural & social sciences ☐ Ecological, evolutionary & environmental sciences

For a reference copy of the document with all sections, see nature.com/documents/nr-reporting-summary-flat.pdf

Life sciences study design

All studies must disclose on these points even when the disclosure is negative.

Sample size	Sample size of 4 was selected as an appropriate trade-off between statistical power and experimental feasibility, taking into consideration the number of treatments and available time and costs. This was not based on statistical pre-analysis, but is a standard choice for this field.
Data exclusions	Two samples were lost in the course of laboratory preparations: One replicate for TAG determination (high-C, no nutrient treatment at 96 hours) and one replicate for PHB (low-C, with nutrient at 96 hours).
Replication	The applied methods are well established in the laboratory and known to be reproducible from previous validation. All measurements were accompanied by analysis of appropriate standards, which were confirmed to be within acceptable bounds of quantification. Further sources of variability between measurements are captured in the variability between replicates and therefore accounted for in the uncertainty estimates of results.
Randomization	Starting soil material was sieved and homogenized before distributing into microcosms, and these microcosms were randomly assigned to treatment groups.
Blinding	Blinding was not applied. Experimenters were only responsible for routine laboratory procedures, with all measurements performed by laboratory intruments (e.g. GC-MS).

Reporting for specific materials, systems and methods

We require information from authors about some types of materials, experimental systems and methods used in many studies. Here, indicate whether each material, system or method listed is relevant to your study. If you are not sure if a list item applies to your research, read the appropriate section before selecting a response.

Materials & experimental systems

n/a	Involved in the study	
☒	☐	Antibodies
☒	☐	Eukaryotic cell lines
☒	☐	Palaeontology and archaeology
☒	☐	Animals and other organisms
☒	☐	Human research participants
☒	☐	Clinical data
☒	☐	Dual use research of concern

Methods

n/a	Involved in the study	
☒	☐	ChIP-seq
☒	☐	Flow cytometry
☒	☐	MRI-based neuroimaging
